# Online Decision Support Tool for Personalized Cancer Symptom Checking in the Community (REACT): Acceptability, Feasibility, and Usability Study

**DOI:** 10.2196/10073

**Published:** 2018-07-04

**Authors:** Marzena Ewa Nieroda, Artitaya Lophatananon, Brian McMillan, Li-Chia Chen, John Hughes, Rona Daniels, James Clark, Simon Rogers, Kenneth Ross Muir

**Affiliations:** ^1^ Division of Management Sciences and Marketing Alliance Manchester Business School The University of Manchester Manchester United Kingdom; ^2^ Division of Population Health, Health Services Research and Primary Care School of Health Sciences, Faculty of Biology, Medicine and Health University of Manchester Manchester United Kingdom; ^3^ Centre for Primary Care, Division of Population Health, Health Services Research and Primary Care School of Health Sciences, Faculty of Biology, Medicine and Health University of Manchester Manchester United Kingdom; ^4^ Centre for Pharmacoepidemiology and Drug Safety, Division of Pharmacy and Optometry School of Health Sciences, Faculty of Biology, Medicine and Health University of Manchester Manchester United Kingdom; ^5^ IT Services University of Manchester Manchester United Kingdom; ^6^ REACT project University of Manchester Manchester United Kingdom; ^7^ Greater Manchester Cancer Vanguard Innovation Manchester United Kingdom; ^8^ Bodey Medical Centre Manchester United Kingdom

**Keywords:** early detection of cancer, cancer education, cancer symptoms, cancer risk, personalized risk, website development, REACT

## Abstract

**Background:**

Improving cancer survival in the UK, despite recent significant gains, remains a huge challenge. This can be attributed to, at least in part, patient and diagnostic delays, when patients are unaware they are suffering from a cancerous symptom and therefore do not visit a general practitioner promptly and/or when general practitioners fail to investigate the symptom or refer promptly. To raise awareness of symptoms that may potentially be indicative of underlying cancer among members of the public a symptom-based risk assessment model (developed for medical practitioner use and currently only used by some UK general practitioners) was utilized to develop a risk assessment tool to be offered to the public in community settings. Such a tool could help individuals recognize a symptom, which may potentially indicate cancer, faster and reduce the time taken to visit to their general practitioner. In this paper we report results about the design and development of the REACT (Risk Estimation for Additional Cancer Testing) website, a tool to be used in a community setting allowing users to complete an online questionnaire and obtain personalized cancer symptom-based risk estimation.

**Objective:**

The objectives of this study are to evaluate (1) the acceptability of REACT among the public and health care practitioners, (2) the usability of the REACT website, (3) the presentation of personalized cancer risk on the website, and (4) potential approaches to adopt REACT into community health care services in the UK.

**Methods:**

Our research consisted of multiple stages involving members of the public (n=39) and health care practitioners (n=20) in the UK. Data were collected between June 2017 and January 2018. User views were collected by (1) the “think-aloud” approach when participants using the website were asked to talk about their perceptions and feelings in relation to the website, and (2) self-reporting of website experiences through open-ended questionnaires. Data collection and data analysis continued simultaneously, allowing for website iterations between different points of data collection.

**Results:**

The results demonstrate the need for such a tool. Participants suggest the best way to offer REACT is through a guided approach, with a health care practitioner (eg, pharmacist or National Health Service Health Check nurse) present during the process of risk evaluation. User feedback, which was generally consistent across members of public and health care practitioners, has been used to inform the development of the website. The most important aspects were: simplicity, ability to evaluate multiple cancers, content emphasizing an inviting community “feel,” use (when possible) of layperson language in the symptom screening questionnaire, and a robust and positive approach to cancer communication relying on visual risk representation both with affected individuals and the entire population at risk.

**Conclusions:**

This study illustrates the benefits of involving public and stakeholders in developing and implementing a simple cancer symptom check tool within community. It also offers insights and design suggestions for user-friendly interfaces of similar health care Web-based services, especially those involving personalized risk estimation.

## Introduction

Cancer is the leading contributor to mortality worldwide; in the United Kingdom (UK) alone there are more than 300,000 new cancers (excluding skin cancers) diagnosed annually, and it is estimated that roughly one-third of the population will develop a cancer in their lifetime [1]. Favorable outcomes are more likely when cancer is detected and treated earlier [2], as noted in the World Health Organization report, “Every year, millions of cancer patients could be saved from premature death and suffering if they had timely access to early detection and treatment*”* [3]. Early detection of cancer could translate into significant savings for health care services, benefiting thousands of patients [4].

Regardless of the relevance of early cancer diagnosis for survival rates, for many years the UK has appeared “near the bottom of international league tables for cancer survival in economically developed countries*”* [5]*.* Hamilton et al [5] attribute this issue to patient and diagnostic delays, which means that patients might be unaware their symptoms could be cancerous and delay reporting it to their general practitioner (GP) or GPs might delay referral to secondary care services. In the UK, the task of early cancer detection typically rests with GPs, who are gatekeepers to all secondary care services [6]. However, “every year, a full-time GP will have one patient diagnosed with each of the four common cancers (breast, lung, colon, and prostate)” [7], thus potential lack of experience with different types of cancers and its symptoms might lead to a delayed referral to secondary care services [7]. Although individuals aged 40 years and over are eligible for the National Health Services (NHS) Health Check, which is “designed to spot early signs of stroke, kidney disease, heart disease, type 2 diabetes, or dementia” [8], cancer is not covered by the program.

To help GPs to minimize the diagnostic delay and expedite referral for diagnostic testing, models have been developed quantifying the severity of different cancerous symptoms in undiagnosed patients [5]. The two models in use with sufficient evidence from systematic reviews showing how those models can improve physician performance are Risk Assessment Tools (RATs) and QCancer [5]. RATs consider only symptoms reported to GPs by patients before a cancer diagnosis from both GP surgeries and electronic medical records in the UK, studying a sample of over 7000 cases involving over 6 million patients [7,9]. QCancer considers both symptoms and risk factors such as age, sex, and cigarette smoking, and is based on medical records of 754 UK general practices [5,9,10]. Both models provide GPs with a positive predictive value (PPV) which reflects the “chance of a patient having the disease of interest when they have reported the symptom” [7]. PPVs can be calculated for a single symptom or a combination of symptoms, can vary from 0.1% to >17%, and the 2015 guidelines of the National Institute for Health and Care Excellence (NICE) recommend further investigation for patients exceeding the threshold risk of 3% [1,5,7].

Considering the effectiveness of RATs and QCancer in improving performance of GPs, as well as the fact that these risk assessment models are still not widely used in General Practice across the UK [5], our objective is to use the information provided by these models and offer it to the public with an aim of shortening the delay in reporting cancer symptoms by patients. It is important to note that RATs and QCancer are different from many existing risk assessment models directed at members of the public. The majority of the existing risk assessment models evaluate individual’s future risk of developing different cancers based on the combination of genetic, environmental, and behavioral risk factors (eg, Your Disease Risk, Reflect) [11,12].

Our focus in this article is on the design and development of the REACT (*R*isk *E*stimation for *A*dditional *C*ancer *T*esting) website, a symptom-based cancer risk assessment tool offered through a Web-based interface in a community setting. REACT is not designed to be a screening or diagnostic tool; but a tool to assist people in deciding whether or not they need to consult their GP about potentially cancerous symptoms. The tool assesses the symptoms of 5 major cancers affecting people in the UK (ie, bowel cancer [also known as colon or colorectal cancer], breast, ovarian, lung, and prostate cancer) and is designed to raise awareness of symptoms that may be indicative of cancer amongst the public. Greater awareness of cancer symptoms could shorten the person’s delay when it comes to recognition and reporting cancer symptoms to primary care, as evidenced by results of some symptom awareness campaigns [5,13-15]. Equally, if the symptom is not found to be related to an underlying cancer then it is important to rule such a possibility out; furthermore, most of the symptoms are sufficiently serious to merit further investigation in their own right. Risk estimation in REACT is based on the RAT models [7]. The reason for using RATs is that, by utilizing a representative record of symptoms and avoiding the complexity associated with a mixture of symptoms and risk factors, it is less susceptible to ascertainment bias when the population studied is not representative of the entire population [5,6].

REACT can help the general public to identify symptoms that may be related to cancers and estimate the personal risk of cancer, as indicated by a PPV. Each clinical symptom listed in the original RAT models (eg, constipation or dyspnea, terms easily understood by GPs but necessarily not by a layperson) was translated into layperson language to be used in the REACT questionnaire. This was done by referring to commonly used descriptors on the NHS websites [16] and other medical sites from well-established organizations, such as cancer charities or government related sites. For example, the Breast Cancer Now organization, one of the UK breast cancer charities [17], was referenced to develop symptom-based questions related to breast cancer. As RATs utilize the most relevant symptoms per cancer (ranging from 5 to the maximum of 9 symptoms) as well as frequency of those symptoms (eg, a single vs reoccurring symptom) [7], we also needed to ensure that reoccurrence of each symptom was recognizable in the questionnaire. This was achieved by adding timeframe for a given symptom (eg, has the symptom occurred more than once in 12 months).

Regardless of the potential of REACT, offering such a tool to the public is associated with various challenges. First, there are individual cognitive and emotional factors that can affect public acceptance of such a tool, and an understanding of these is necessary for successful development and approval of internet-based health interventions [18,19]. As cancer is one of the most feared diseases [20-22] and a cancer diagnosis is often life changing [23], individuals may avoid considering this issue by downplaying their own cancer risk or ignoring symptoms. Therefore, any intervention offered to the public should aim to minimize potential anxiety, at the same time showing early cancer detection as a positive step to improve treatment potential. While the existing literature does not show that risk assessment communication increases anxiety [11], it is important to consider risk communication literature in developing risk assessment tools that are likely to minimize potential fear associated with the communicated risk [24,25]. Thus, it is important to understand how to increase perceived self and response efficacy (ie, belief one can perform the recommended actions such as discussing REACT results with his or her GP and belief that results of such a discussion could minimize the threat), factors shown to have mitigating effects on fear experienced during risk communication [24,25].

In addition, when designing such a tool, the optimal (leading to best understanding of the risk score) approach to communicate personal risk to individuals is essential. While there is plethora of recommendations on how to communicate risk (eg, presenting outcome estimates, using visual formats, using evaluative labels about estimates) [26,27], more research is needed on how to combine those different recommendations in actual symptom-based intervention about cancer.

Furthermore, the user needs in human-computer interaction involving a website need to be considered. For instance, website content, layout, look and feel, may all affect perceived usability [28-30]. There is also a need to consider different stakeholders that could utilize the website in the future (eg, community pharmacies, NHS Health Check teams) and use their views to understand the best implementation pathway for such an intervention.

## Methods

### Overview

Our research utilized focus groups and open-ended questionnaires distributed firstly at a showcase event dedicated to development of the REACT website, and secondly during a trial of REACT within community settings. Six focus group interviews preceded with a trial of the website involving the “think aloud” technique [29,31] were conducted with members of public and health care professionals (who could potentially use REACT in the future) in Greater Manchester, UK. Furthermore, the research team organized an event showcasing REACT to members of public. Participants of the event were invited to participate in research, use the REACT website, and provide their feedback in an open-ended questionnaire [32]. The same open-ended questionnaires were also used in a trial within community settings where members of the public could fill in the REACT questionnaire with assistance of a health care professional.

**Figure 1:**
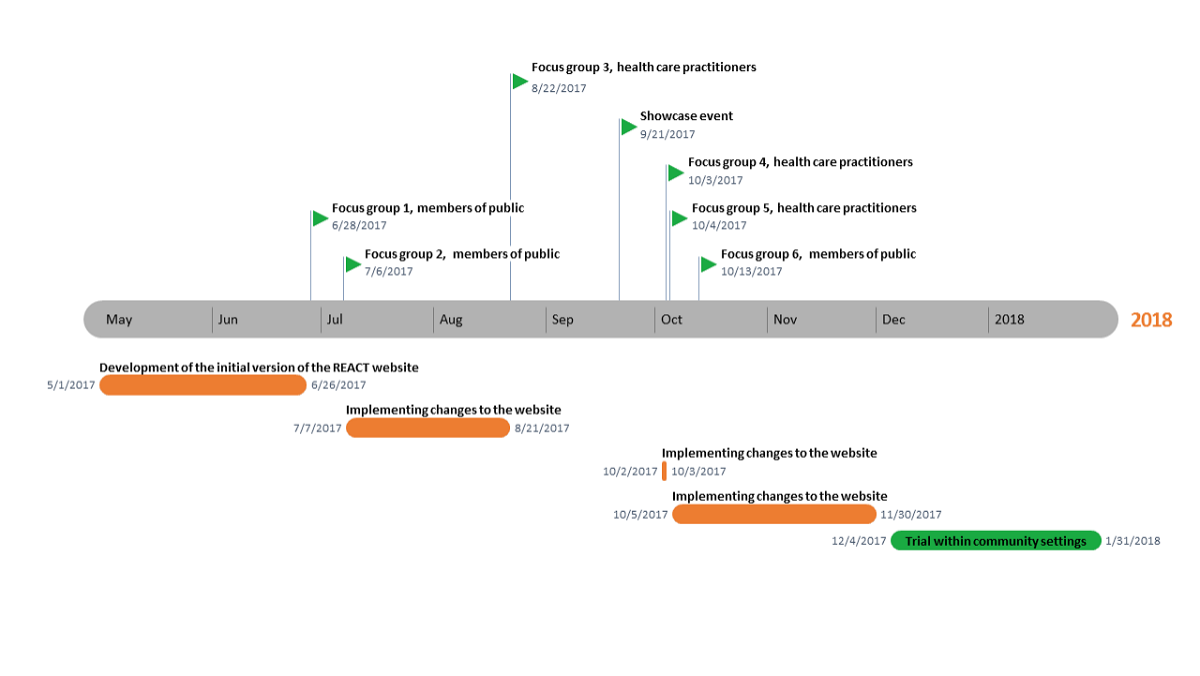
Timeline of research into the REACT (Risk Estimation for Additional Cancer Testing) website.

Data were collected between June 2017 and January 2018, with a timeline for each research step illustrated in Figure 1. Participant recruitment, data collection, and data analysis continued simultaneously, allowing for website iterations between different points of data collection and check for acceptability of the modifications in subsequent stages of data collection. The majority of changes to the website were implemented between July 7 and August 21 2017, and between August 23 and October 2, 2017. Following the focus group on October 3 2017, only minor changes were implemented to the website as the feedback gained in those studies predominantly featured themes and ideas previously mentioned by research participants, thus pointing to data saturation [33]. The issue of data saturation was also discussed and agreed upon by members of the steering group overseeing the research project, meeting on a monthly basis. Ethical approval was obtained from the University of Manchester Ethics Committee (Ref: 2017-2065-3599). All participants participated in the research voluntarily and provided written consent.

### Design

Our research into REACT involved 6 focus groups with 15 members of public and 20 health practitioners, and 24 open-ended questionnaires with self-reported evaluation of the REACT website provided by members of public.

Three focus groups involved members of public and 3 involved health care practitioners. The “think aloud” technique was used to explore users’ website experience. Qualitative research, especially involving “think aloud” procedures, is considered the most appropriate method to gain insights about user perceptions and for usability testing of websites [29,30,34]. The “think aloud” technique relies on research participants reporting their experiences, thoughts, and ideas while using the website. This approach addresses the issue of data loss which can be experienced when information is collected after website use. This technique typically leads to identification of between 80% to 90% of usability problems of an evaluated website [35-37]. Focus groups were preferred over one-to-one interviews as they allow for views and opinions to be developed and discussed, at the same time allowing for reporting of individual opinions [38].

Apart from focus groups, we also utilized open-ended questionnaires enabling self-reporting of user experiences without the presence of a researcher, often referred to as asynchronous remote usability evaluation [39]. Such techniques are deployed due to the benefits of collecting usability data from many participants in a relatively short amount of time [39]. Use of open-ended questionnaires is also a technique used in market research for the purpose of evaluating consumer and user views about a product or service [32]. Apart from being easy and convenient to implement, this technique is appreciated for its ability to elicit spontaneous views and less prone to response bias [40].

### Participants and Recruitment

Focus groups one, two, and six were made up of members of the public. Focus groups three to five involved health care professionals. Members of the public (men and women) aged 40 years and older, with or without a previous history of cancer, and with a range of backgrounds were invited to participate in the 3 focus groups. We targeted people who could potentially use REACT in the future. Individuals with a history of cancer were not excluded, as (1) having cancer does not mean someone will not develop another type of cancer in the future; and (2) those individuals could evaluate the symptoms they had experienced and raise issues in the event that the REACT algorithm was not accurate. Our sampling objective was to obtain diverse representation of views, not to compare views of different groups of people. Focus groups participants were recruited through Macmillan Cancer Support, via posters around the university and community centers within the Greater Manchester area (eg, gyms and libraries); and from the NHS Cancer Bowel Screening Program. Participants were reimbursed for their time with a £20 high street voucher except for two participants who did not accept it. Participant characteristics are summarized in Table 1.

In addition, health promotion or prevention professions that could in the future take an active part in implementing REACT into health services ecosystem in the UK were invited to participate in 3 focus groups. Participants were (1) employees of a community pharmacy (n=10), (2) NHS Health Check workers within Greater Manchester (n=5), and (3) members of the NHS Cancer Prevention and Early Intervention group in Greater Manchester (n=5). These focus groups were organized as part of presentation sessions about REACT to the Clinical Commissioning Groups, where GPs and other health professionals were present.

**Table 1:** Sample characteristics of members of public (focus groups and open-ended questionnaires combined).

Characteristic		Value, n (%)
**Age (years)**		
	40-49	8 (20)
	50-59	12 (31)
	60-69	9 (23)
	70 and over	4 (10)^a^
**Gender**		
	Male	20 (51)
	Female	18 (46)^b^
**History of cancer**		
	Yes	6 (15)
	No	33 (85)

^a^Six participants did not reveal their age.

^b^One participant did not reveal their gender.

Participants evaluating the REACT website through open-ended questionnaires were recruited through (1) an event organized by Greater Manchester Cancer Vanguard (GMCV), the founder of research into REACT, and (2) a trial of the REACT website within community settings. The showcase event was a presentation of research undertaken into REACT and was open to the public and GMCV associates (patient groups, health care representatives, and industry), and was advertised through various channels, such as the GMCV website, newsletters, social media channels, and email. The event was attended by 32 participants and 10 of them agreed to provide evaluation of REACT through an open-ended questionnaire.

As the feedback from all the data collection pointed to use of REACT in assisted manner, with a health care professional present, we also evaluated user experiences during a trial of REACT in community settings. A local community pharmacy agreed to recruit participants (pharmacy customers) and assist them with filling in the REACT questionnaire. Following the evaluation, participants were asked to provide their feedback through the use of the same open-ended questionnaires used after the GMCV showcase. A total of 14 questionnaires were collected during that research phase.

### Data Collection

The focus groups were conducted by 3 moderators—female researchers with experience of conducting qualitative research (a market researcher, an epidemiology researcher, and an academic clinician)—as well as the software engineer responsible for the design of the REACT website and a note taker. Each focus group lasted between 60 and 90 minutes. Most of the focus groups were performed in a room at the University of Manchester, and 2 focus groups with health care practitioners were performed at the participants’ workplace. Field notes were taken in each focus group [41].

The user experiences of REACT were collected during a trial of the website by using the “think aloud” technique [29]. During the “think aloud” procedures participants in each focus group were split into smaller groups of 2 to 3 participants, with each group accompanied by one of the focus group moderators. This separation was aimed at obtaining independent views, unbiased by the influence of the majority of the focus group participants.

After the trial of the REACT website, participants were asked (after merging into one group) a series of questions about different pages and sections of the website (eg, landing page or cancer questionnaire page). The questions about different pages were accompanied by a screenshot from that page.

Finally, participants were asked about their general impressions of viewing and using the website within the health care service (eg, on their own or with the help of health care practitioner). Depending on the area of expertise (ie, members of the public or health care practitioners) emphasis was placed on different questions. For instance, members of the public were questioned more about the user experience than the practitioners were; and the opposite was the case for the questions about potential implementation of programs such as REACT within health care services.

The link to open-ended questionnaire was emailed to the participants of the GMCV showcase who had previously agreed to participate in research. Those individuals were provided with a temporary link to the REACT website (available only to those participants for a week) and they were asked to use the website and to fill in the open-ended questionnaire. The questions asked in the questionnaire are provided in Multimedia Appendix 1. The same questionnaire was later used in the trial within community settings, when research participants filled in the questionnaire after completing the REACT assessment in their community pharmacy.

### Data Analysis

The interviews were audio-recorded and transcribed verbatim by a professional agency. The data obtained from open-ended questionnaires were exported from the survey software into a Word document. Data were analyzed with a thematic analysis approach [33]. Four researchers (the same group that was involved in data collection procedures) read all the documents and searched for patterns in the data, focusing on the patterns that related to research questions and objectives of the study.

The coding of usability evaluation data was guided by 2 different perspectives: (1) navigation strategy, pointing to used navigation tools, and (2) navigation problems (or facilitators), pointing to potential barriers (or facilitators) to completing the cancer risk evaluation activity [29,42].

The data about website design were coded into 2 main categories: (1) website design and (2) risk presentation. In the website design section, 3 factors were identified: content and related functionality, readability of the cancer questionnaire, and website look and feel. In the risk presentation section, we identified problems about different types of risk presentation: numeric, visual, and evaluative. The attitudinal and perceptual data about REACT and its future implementation into health care services were coded by highlighting existing cognitive or emotional states as well as barriers and facilitators to implementation.

The initial analysis was performed by 4 researchers (the same group that was involved in data collection procedures). The coders showed fairly high levels of consistency in coding the themes, with kappa statistics between 0.71 and 0.87 [43]. The final validation of the results was performed by members of the steering group overseeing the research project.

## Results

### Overview

Sample characteristics for the members of public group are provided in Table 1. Practitioners participating in this research held various positions, including pharmacists, pharmacy senior management, NHS Health Check workers and management, a multi-agency group, inclusive of a GP from the NHS in Greater Manchester, and cancer awareness facilitators. Of the 20 participating health care practitioners, 13 were female.

In general, the feedback in relation to the website and its implementation into health care services was consistent across members of the public (regardless of whether they had cancer history or not) and health care practitioners. Therefore, the views of these groups are summarized together. In cases where the rationale for certain preferences differed between the groups, the differing views were elaborated.

Finally, the view of the majority of participants was that REACT should be offered in community settings with assistance of a health care practitioner and individuals should not attempt to assess their risk on their own. While we discuss the details on pathways to delivery of REACT later, it is important to mention this issue before discussing the results in more depth, as some of the comments relate to assisted delivery of this service.

### Perceptions and Attitudes in Relation to REACT

The initial reaction to REACT was very positive, with all participants appreciating the value and importance of such an intervention. While some members of public were aware of symptoms of specific cancers (eg, bowel cancer), in most cases, their awareness of different cancers and its symptoms was limited. Hence, they appreciated the capabilities of REACT. As one participant stated:

I think it’s important to be informed and to get there as early as possible. To go and see the GP, and if there’s a tool perhaps, you know, to help me do this, that may be of interest.

Focus group 2

One of the greatest challenges seen about the future of such a tool was related to creating a positive image of this intervention. This was important, considering the perception and stigma of cancer as a deadly disease. Participants reported that it was of utmost importance to emphasize the positive aspects of early detection tools, and their relevance to cancer management and improving survival rates. This was emphasized by the following quotes from participants:

I don't think it's an issue with leaflets [promoting early cancer detection]. I think it's an issue with cancer. And I'm wondering whether there is a way to raise awareness of cancer symptoms without stigmatizing it.

Focus group 3

Advertise it [REACT]. Social media is probably quite a good one because it's being increasingly used not just by young people, I mean, things like Facebook seems to be…I mean, a lot of the youngsters are a bit like, oh, I've gone off Facebook, I don't use that anymore. But my generation seem to be more and more into this…

Focus group 1

Some participants suggested using testimonials from cancer survivors or celebrity endorsement to promote early detection and the role REACT can play in it.

Also on here could be, you’ve got statements here [landing page] about people who did the assessment and did not have cancer. It would be really nice for somebody to say: “So I did the assessment and it really put my mind at rest.”

Focus group 3

It helps to get a celebrity endorsement, I think, you know, a celebrity that's had cancer or something.

Focus group 1

**Figure 2:**
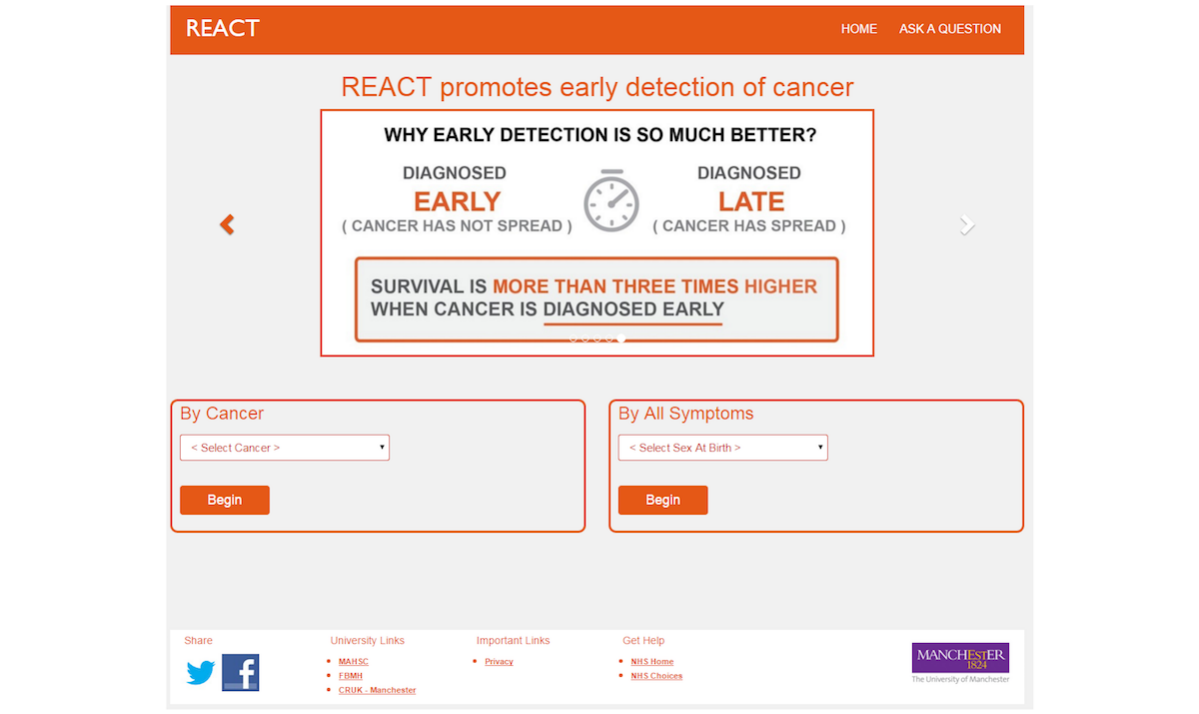
Image on the REACT (Risk Estimation for Additional Cancer Testing) website promoting the importance of early detection of cancer.

Following those suggestions, the content of the REACT website emphasizes early detection success rates (Figure 2, content based on a graphic created by Cancer Research UK) and also contains individual testimonials at the carousel (changing display) available on the landing page.

### Key Features of a User-Friendly Website Design

Participants identified some key features related to a user-friendly design of the website, which can be classified into 3 key categories: content and associated functionality, clarity and readability of the cancer questionnaire, and look and feel of the website.

#### Content and Functionality

Content and related functionality of different sections of the website were crucial in affecting perceived usability and user-friendliness. Originally, the REACT website only allowed users to evaluate their symptoms in relation to a particular cancer (eg, breast cancer). However, the participants, especially health care practitioners, regarded it was important that the website allows for 2 methods of completion, either by cancer type or by symptom. For example, one focus group participant noted:

I think… firstly we have to select which cancer you want to check, as this is something I do not think many of our customers will know. So, you need to help them here and list various symptoms for different cancers.

Focus group 3

Consequently, the revised version of the website allows users to select a questionnaire either by cancer type or by symptoms (Figure 2). If someone selects the questionnaire by symptoms (vs a single cancer type), they will answer more questions as there are more symptoms related to different body parts to be evaluated.

The cancer symptom questionnaire (described in the following section), and the information it provided to individuals, was regarded as another content factor that affected the perceived functionality of REACT. Participants indicated that apart from obtaining their risk estimation after trying REACT, they also wanted something more tangible about that risk score that could serve as a decision aid in a potential meeting with a health professional. For example, one focus group participant asked:

How do I know what affected my risk score? And how do I communicate this information to my GP?

Focus group 1)

Health care practitioners also supported that idea, but for them the tangible output of the risk assessment was considered as an important factor that could simply improve customer journey for their clients or patients.

I think for me the thing that will determine how we use it (REACT) here, is that customer experience, so that they come out of that discussion knowing what to do next, where to go but ultimately they’re not walking out of that room suicidal about their result. So, we need to make sure that they go out with the right information feeling positive about taking that test. So that customer journey is really important.

Focus group 3

Consequently, REACT provides individuals (and their GPs via email) with a summary of the symptoms that triggered the risk score and signposts them to various support resources (eg, the Cancer Research UK website as an example of an educational resource or encouragement to contact own GP as an example of a more actionable behavior pathway). This information can help individuals to recognize and understand cancer symptoms better and can be a tangible decision aid supporting decision making during a consultation with GP, thus rising perceived self-efficacy and minimizing anxiety associated with risk communication [24,25].

Furthermore, some participants inquired whether it was possible to obtain any further cancer-related information from the website, especially in case the symptoms they evaluated with REACT were not associated with a higher risk of having cancer. Some individuals felt fortunate that their symptoms were not due to cancer, but still wanted to find out whether there was anything they could do to minimize any future risk. Health care practitioners considered the information on how to reduce future risk of having cancer as a value-added factor that could contribute to that important consumer experience. For example, one focus group participant noted:

Is there anything those people can do to reduce their future risk of having cancer? If there is something like that, it just could be worth the extra time [guiding people through another questionnaire] if we can improve the customer journey…

Focus group 3

To address those requests, after displaying an individual’s risk estimation on the results page (after filling in the cancer questionnaire), REACT provides a link to a website that deploys risk algorithms to calculate individual risk of having cancer in the future: REFLECT (*R*isk *E*stimation *F*or *L*ifestyle *E*nhancement *C*ombined *T*rial) [12]. If interested, users could learn about how their lifestyle choices (eg, smoking and physical activity) affect their future cancer risk and can see how potential lifestyle changes would affect their cancer risk. Provision of a link to REFLECT was seen as a way to increase perceived response efficacy and minimize anxiety related to risk communication [24,25].

In addition, some participants suggested that the website should include an explanation of how REACT was developed, emphasizing the scientific background, expertise, funders, and stakeholders that contributed to the website development. This is currently addressed by designing an “About us” and “News” section of the website.

#### Cancer Questionnaire

The cancer questionnaire, in particular its clarity, readability, and ability to point to relevant symptoms, was of utmost importance to participants. In general, participants were in favor of language that could be easily understood (eg. explain that diarrhea relates to symptoms such as loose or watery feces and/or stomach cramps). As research has shown that keeping some level of medical terminology enhances the credibility of a website [44], in cases where medical terminology was used, a layperson explanation was provided where possible. Our changes were well received by research participants:

One thing I like about the tool is that it’s really easy-to-use, I think, no matter how confused or illiterate, I don’t think anyone would struggle with the yes/no and the tapping what your answer is. Besides, one of us [health care practitioner] would be most likely there to explain any questions, right?

Focus group 4

One way to reliably assess readability of written material is to use tests for readability [45,46]. The wording in the questionnaire, as well as other parts of the website, was adjusted using the Flesch Reading Ease score to ensure readability scores were 60% and above (out of 100%, where a score between 60-70% translates to a UK or US grade 9-10 or 8-9 respectively, when students are 13-15 years old) [46,47]. Cancer questionnaires (for single cancers and for symptoms) have the highest readability scores, with readability ranging from 72% to 88%, risk results have the lowest readability scores ranging from 60% to 70%. Thus, the REACT content shows satisfactory readability levels.

Another aspect related to the ability of the questionnaire to highlight actual changes in one’s body that would point to potential cancerous symptoms. An example of this problem is the fact that some of the symptoms in the questionnaire might be “normal” for some people (eg, loose feces or bloating existing throughout one’s life), and hence might produce an overestimated risk score. The following quotes from focus group participants illustrate this issue:

You know, when you explain that diarrhea can be associated with going more frequently to the toilet … although there are more detailed symptoms below, my first impression it that I might answer ‘yes’ but this will be because of my diet… I drink a lot of water and have a fiber-rich diet.

Focus group 6

I might be bloated because I have IBS [Irritable Bowel Syndrome]) or because I am a female, and we older ladies can be like that…So would I select it as a symptom? I know bloating is an important one for ovarian cancer that is often missed.

Focus group 1

To address this issue, the following sentence was added at the start of the questionnaire: *“*When you answer the questions, please try to think about symptoms that are not normal for you*”.* This addition was designed to help individuals avoid pointing to symptoms that were unlikely due to cancer. While this could pose danger of omission of an important symptom, the fact that health care practitioners would be able to assist during the assessment would solve this potential problem.

**Figure 3:**
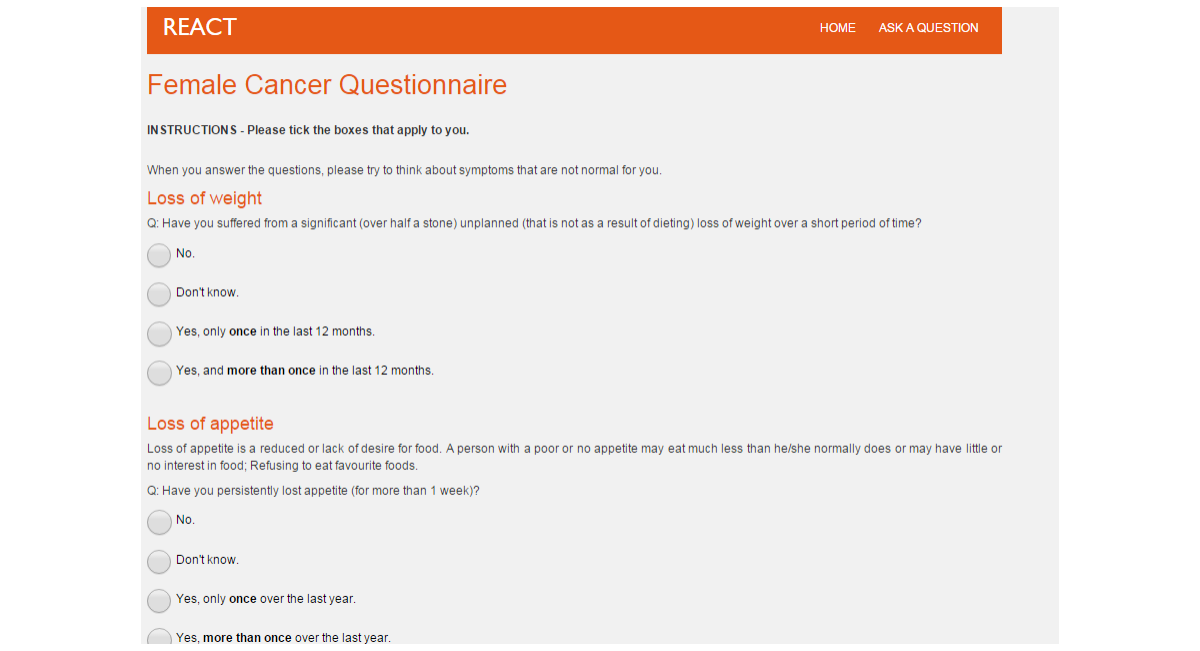
An example of cancer questions.

Furthermore, building on the ability to distinguish relevant changes in one’s body, an important aspect was to ensure that the questionnaire provides all possible options for potential answers (eg, “Yes,” “No,” or “Do not know” in relation to a symptom such as bloating). In case a symptom was newly appearing, it was also important to emphasize the difference in frequency of experiencing that symptom, as well as how recently they had that symptom (Figure 3).

#### Website Look and Feel

Firstly, it was important that REACT allowed users to easily access the cancer questionnaire. The initial versions of the website had a short introduction page explaining what REACT was and what it did. This was followed by a disclaimer, then the questionnaire. However, participants did not like the “waiting” and “clicking” associated with getting to the questionnaire, for example one focus group participant noted:

Yeah, just one disclaimer… But I mean by the time I've read through this I have lost interest. I mean I've read a lot on the first page, I've seen all of this but I've had enough, you know, I'm going to go and watch TV or, you know, go and make a cup of tea, or something.

Focus group 2

**Figure 4:**
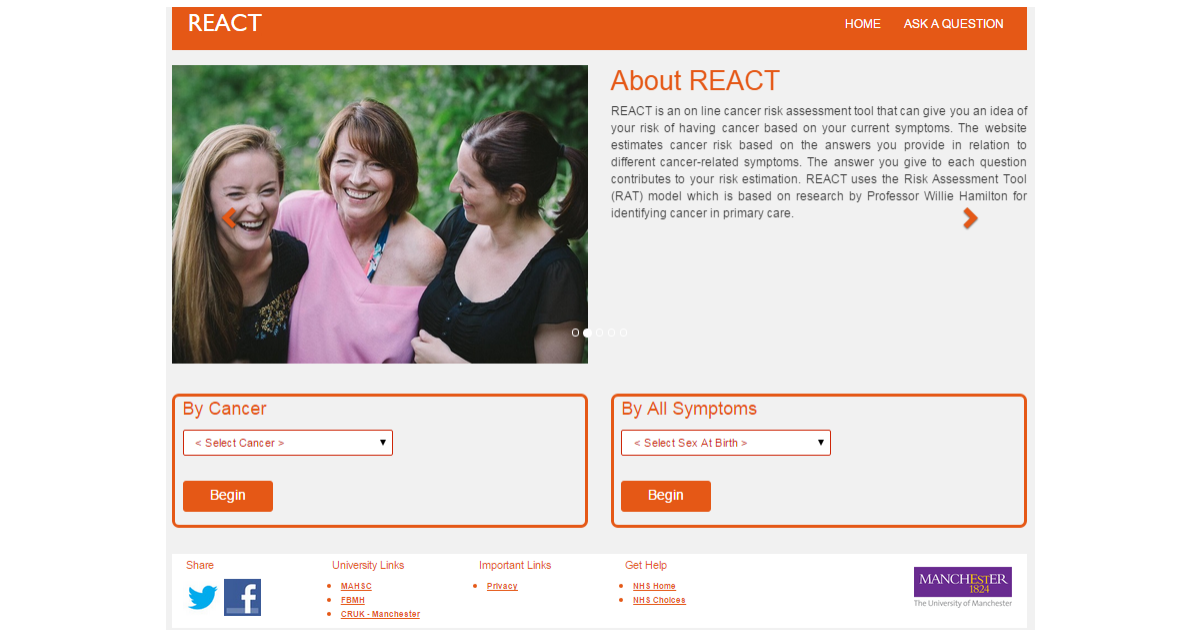
Look and feel of the REACT (Risk Estimation for Additional Cancer Testing) landing page.

Consequently, the REACT landing page incorporates an introduction and prominent “start” button, followed by a succinctly phrased disclaimer (Figure 4). To provide different types of information at the landing page, without stopping participants from starting the questionnaire, we provided REACT related information, testimonials, and early detection information (Figure 2) on a carousel, a moving display that changes images every 20 seconds but also can be changed instantly by clicking on the arrows.

An important decision about the website design related to the selection of the main color used on the website. Because different colors have been associated with reflecting emotions and personality types and are often used by different organizations and brands to reflect organizational or brand values [48,49], we wanted to use a color that would be encouraging and invite participation. Originally, we used green, which is often related to health and nurturance [49]. However, this was criticized by some respondents as too “cheerful and relaxing” in the context of early detection of cancer. Consequently, we used orange, which is perceived as warm, optimistic, and sociable [49,50]. This color was well received by research participants who often mentioned (unprompted) the color of the website as one of the most likeable and noticeable website features.

Another important observation related to the fact that participants wanted the website to have a “human” image rather than one that is more medical or technical. One way to assure this was to use some images to reflect human values and lifestyles. This proved to be a challenging task. At first, we used cartoon images, which were quickly criticized by participants as inappropriate for the target audience. The following quotes from focus group participants illustrate the concerns with using animations on the website:

And then the second thing [animation] that came after is that this is for families when, you know, a lot of people, they're likely to be older; they're likely to be individuals living on their own. I don’t see any connection with that image in cancer other than a kid’s drawing of their family. So, I don’t like it.

Focus group 2

I would use animations when sending a WhatsApp message to my kids or something like that. On there, basically, you only do it for a bit of humor to add to things. Seeing it on there, I don't think it's doing any harm, but it's not helping me.

Focus group 1

Therefore, the animations were replaced by real-life images (Figure 4), which were more favorably received. The images were of real people, not posed stock images (disliked as “too perfect”), and this was appreciated by participants, as it contributed to creating a realistic image of REACT as a community tool. Health care professionals emphasized the need to make the website appealing to different ethnic groups. This issue of social inclusion and exclusion was considered a very important and challenging outcome to achieve:

We often struggle to reach out to different ethnic groups. We for instance work with Bangladeshi females, who… my guess is, would not feel REACT is designed for their community - based on the images you have here. One way would be to make sure your images reflect that diversity… But to capture this you probably need to hire a photographer and work with them within communities. So that what you get is realistic. But there is no perfect solution.

Focus group 5

Following this suggestion, more diverse images featuring people of different races, ages, and ethnic groups were added to the carousel on the landing page.

### User-Friendly Risk presentation

Risk presentation proved the most difficult aspect in developing REACT, as people wanted to ensure the risk score was understandable and at the same time motivating (serious) enough to lead to action. In general, participants preferred a simple frequency format of risk presentation (eg, x in 100) over percentages (eg, x %). Furthermore, they felt more comfortable with having their risk information provided in both written (numeric) format and a visual format.

**Figure 5:**
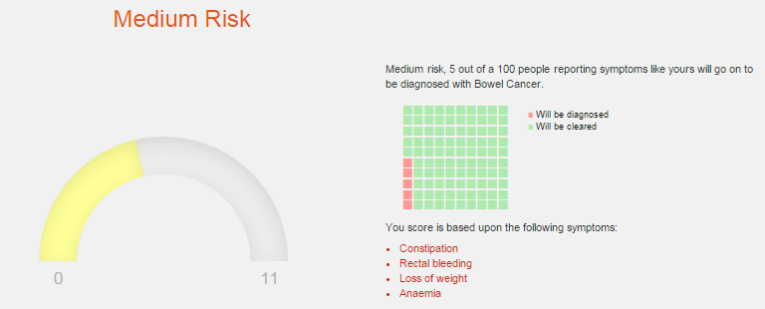
The selected graphics for individual risk presentation: a graph illustrating one’s risk in relation to affected individuals at risk (left) and a “waffle chart” illustrating one’s risk in relation of the entire population at risk (right).

Interestingly, when the risk estimation was presented as a graph, participants preferred to have the graph representing one’s disease risk in relation to affected individuals (as illustrated on the left side of Figure 5; in this case, the score would be 5 out of 11, where 11 refers to number of people with the same symptoms diagnosed with the disease). When graphs were used as reflective of the entire population at risk (following the same example discussed above with 5 people, this would be reflected as 5 out of 100 people with symptoms like yours, majority of whom were not diagnosed with the disease, as only 11 were), this made an impression of a very small risk. However, the visuals with the entire population were still desirable, and favorably received if presented as icon arrays (blocks or stick figures such as the “waffle chart” visual used in REACT, as illustrated on the right side in Figure 5).

Regarding the evaluative labels about personalized risk estimates, participants were satisfied with a distinction between low, medium, and high risk. Yellow was used to show low and medium risk, and red to illustrate high risk. Those decisions were again guided by feedback that having any of the symptoms would increase anxiety, and also can signal another disease. In earlier versions of the REACT website, green was used instead of yellow to point to low risk, but while this was liked by members of public, it was discouraged by health care practitioners. For members of public, green was seen as a “safety zone”, assurance that the symptoms are not cancerous. However, health care practitioners saw the “green light” option as potentially leading to complacency in case of non-cancerous symptoms that could signal another illness:

So, people may be using this for cancer, but they may not present with any symptoms of cancer at all, but they may present with symptoms of diabetes…and the “halo effect” of that [green light] can be dangerous.

Focus group 3

To minimize unnecessary anxiety, the meaning of the score was explained with the following sentence:

Remember, most people with this result will not have cancer. Even if you are one of the small number that turns out to have cancer you have done the right thing by completing the questionnaire and going to see your GP, as a cancer discovered early is much more likely to be easily treated, and is more likely to be curable.

message for medium risk

As the data collection process allowed for website iterations between different data collection sessions, the process of website changes is illustrated in Table 2.

**Table 2:** Development stages for Risk Estimation for Additional Cancer Testing (REACT).

Time	Development and changes to the REACT website
May 1st to June 26th 2017	Development of the initial version of the REACT website
July 7th to August 21st 2017	Simplifying the website (eg, giving immediate access to the questionnaire, changing disclaimer to a single click pop up) Change of website theme color from green to orange Removal of any animations included on the website Addition of realistic images with people Adding carousel to the website (including user testimonies, REACT description) Cancer questionnaire iterations Cancer risk presentation iterations
August 23rd to October 2nd 2017	Addition of multiple cancers Enabling printout for users pointing to the cancerous symptoms Addition of the REFLECT model Cancer questionnaire iterations Cancer risk presentation iterations (mainly added “waffle chart” visual for representation of 100 people representing population risk) Positive framing in relation to early cancer detection (mainly in the carousel on the landing page of the website)
October 5th to November 30th 2017	Cancer questionnaire iterations (emphasizing that the questions relate to “not normal to you” symptoms Cancer risk presentation iterations (using both affected and population risk presentation)

### Pathways to Practice

As indicated earlier, and illustrated throughout the reported results, research participants indicated the preferred methods of delivering and receiving advice from REACT. While some participants believed that the advice could be offered to public (via a public website), the majority believed the best way forward was through a guided approach supported by health care practitioners. This type of delivery was strongly recommended by health care practitioners. As cancer is an emotive topic, it was believed that while some individuals could cope with the results they obtained, others could experience stress and anxiety about their results. Considering the fact that some individuals might need help with interpreting and answering the REACT questions (eg, what is normal to me and how do I report it in the questionnaire) and results (eg, I have a low risk of cancer but I am still concerned about the symptom(s) I had reported), as well as using technology, it was believed that they might need professional help in order to complete and understand their cancer assessment correctly. Considering this feedback, it was not recommended that REACT or similar websites are available in the public domain.

Participants believed that REACT can be offered at a variety of locations, where the needed support can be offered. Apart from community pharmacies and NHS Health Check services, respondents who filled in the open-ended questionnaire indicated that REACT would be a desirable addition in voluntary organizations (87% agreed), leisure centers (79%), council offices (75%), and workplaces (75%), to a lesser degree in benefit offices (46% agreed this was a good idea).

## Discussion

### Overview

In this study we provide novel insights into how members of public and health care practitioners perceive a Web-based intervention providing personalized symptom-based cancer risk estimation. We report the process of developing such a website and evaluate opportunities for introducing such an intervention within health care services in the UK. Our results show that there is a need for such a tool and that it would be well received. The best way to offer it to the public appears to be through a guided approach, where a trained individual supports members of the public in the process of risk assessment and evaluation.

Website design, content, functionality, look and feel as well as risk evaluation and presentation are all important factors affecting perceptions of usability of such a website. Users want a positive image of early cancer detection, a simple website with the ability to evaluate and detect multiple cancers, and value added in the form of explanation of their risk score and further lifestyle-based information about potential reduction of their future risk of having cancer.

While medical content and sources of information on such a website are important, individuals want those tools to have a “human face” and community feel that can be conveyed by the use of real-life images of people from various backgrounds and links to social media, community groups and portals. In terms of risk presentation, a direct approach to cancer communication is preferred. All risk presentation types (ie, numeric, visual, and evaluative) are appreciated, with two types of visual risk representation desired: with affected individuals only and the entire population at risk.

### Comparison with Prior Work

#### Perceptions and Attitudes in Relation to Community-Based Cancer Risk Estimator

Our study is the first to date to demonstrate the potential of offering a symptom-based risk estimation tool in relation to a current cancer diagnosis (and potentially other diseases) to the wider public in the UK. Taking such a tool, typically used only in Primary Care settings [5,7,10], and offering it to public in a guided way might be a way to address the issue of patient and diagnostic delays which are often seen as barriers to early detection of cancer [5]. In addition, this research shows that individuals show willingness to understand their symptoms better and appreciate the ability to subsequently verify if those symptoms could be cancerous. It appears that such a community-based approach can be a starting point to shared decision making in the field of health care [51,52].

#### User-Friendly Website Design

Our findings confirm the growing need for Web-based tools like REACT that could facilitate shared decision making, and in the case of cancer, lead to early detection [51,52]. Users desire such tools to help overcome the stigma of cancer by being encouraging and by emphasizing positive, gain-framed outcomes [53]. Furthermore, the results indicate the need to emphasize the scientific evidence for REACT and show the need to build associations of expert knowledge and trustworthiness in relation to this new intervention, noted previously as important in health-related websites [28,44].

Apart from the image of the website, a functional yet at the same time simplistic design is crucial, as noted earlier in literature [29,51]. In the case of REACT, functional aspects include the ability to cover multiple cancers and clear readability of the cancer questionnaire and associated results [51]. Creating an engaging, realistic, and socially inclusive look and feel of the website is crucial in successfully promoting such interventions to a wider public [28,54].

#### User-Friendly Risk Presentation

In general, the research findings in relation to risk presentation show that a range of numeric, visual, and evaluation risk estimates can provide most value to different users with different numeric skills [26]. Interestingly, the participants have shown some preferences in relation to each type of presentation. The preference for simple frequency (eg, x in 100) over percentages (eg, x %) might point to the fact that risk presented as a simple frequency is perceived as higher [55,56]. Such preferences are consistent with the preference for a direct approach to risk communication indicated by research participants.

Considering different types of visual presentation of data and different preferences [26], an option of showing two different visuals appears as the best option. Consequently, while we follow recommendations of Garcia-Ratamero and Galesic [57] in ensuring that a visual with the entire population at risk is shown to REACT visitors, we also follow recommendations for visual risk presentation for greater risk aversion, observed with a graphical display showing only the number of people affected [58,59]. Using two different graphs and assistance of a trained professional present during taking the REACT questionnaire can help to clarify potential confusion in relation to those different risk displays. Showing individual evaluative labels for their risk score (ie, low, medium, or high risk) has been recommended to help users understand their personal risk in the context of the disease [26].

Finally, while REACT does not manipulate fear appeal to affect behavior change, it’s undeniable that communicating cancer risk estimates is associated with certain amount of fear for REACT users [24]. Thus, our findings (although relating to constantly present rather than manipulated fear appeal) can advance the risk communication literature [24,25] by pointing to ways to increase self and response efficacy when communicating symptom-based cancer risk. Providing people with clear printed information about specific symptoms is seen as an important aspect that increases their confidence that they can successfully describe their symptoms to their GPs, thus raising their self-efficacy. Response efficacy in this case mainly relates to breaking with the stigma of cancer as *only* a deadly disease. To help people feel that talking to their GP about their symptoms can help them improve their health outcomes, it is important to make them aware that cancer diagnosed early is more likely to be treated successfully. In this case, early detection should be seen as a way to reduce the risk of late cancer detection when cancer has spread and is more difficult to treat [60].

#### Pathways to Practice

The opportunities for using tools such as REACT are considerable and all the more important in the current financial climate when resources are scarce [61]. The tool has been shown to be user friendly, helpful and empowering. It has the potential to be used in the health care setting, alongside other health related activities such as the NHS Health Check and NHS screening programs as well as part of other consultations such as a chronic disease review. There is scope for the tool to be used in the voluntary sector with trained volunteers helping people, such as the elderly, people with a disability, those accessing community venues or Black and Minority Ethnic groups to know more about their health and potential cancer symptoms.

The biggest opportunity, albeit also with some risk, is for open access to the tool perhaps linked to an existing NHS website such as NHS Choices, where a positive result leads to an automatic referral and triage by a health professional and potential direct referral to diagnostic services. The risks with this include multiple referrals of the “worried well” and increasing the strain on limited resources. The opportunities include earlier diagnosis and faster access to treatment for an audience who might feel more comfortable entering symptoms into an online questionnaire, rather than speaking to their GP.

### Limitations

The results of this study into a wider context need to be interpreted with consideration of the limitations. First, this research is limited to the context of the UK health care system and the 5 evaluated cancers. Consequently, future research can evaluate different cancer types and as the number of questions in the REACT questionnaire increases, further work using cognitive techniques [62] will be needed to further refine question wording and response options. Furthermore, due to differences in health care systems across the world, implementation pathways for interventions like REACT might be different for different countries.

Second, participants representing members of public were a small and self-selected group of individuals. This means that our sample could be limited to individuals who show a strong interest in their health or even in (avoiding) cancer, especially considering the fact that some were previously diagnosed with the disease. While we tried to address this limitation and reach a diverse group of participants by recruitment at various locations and using different means, we cannot exclude the possibility that self-selection impacted our results. This issue will be further explored in the next stage of this research project involving an evaluation of REACT. This future evaluation will consider the ability of REACT to detect symptomatic patients as well as its impact on GP workload, secondary care referral rates, and impact on health economics.

## Appendix

Multimedia Appendix 1 REACT open-ended evaluation questionnaire.

## Abbreviations

GP: general practitioner
GMCV: Greater Manchester Cancer Vanguard
NICE: National Institute for Health and Care Excellence
NHS: National Health Service
PPV: positive predictive value
RAT: Risk Assessment Tool
REACT: Risk Estimation for Additional Cancer Testing
REFLECT: Risk Estimation For Lifestyle Enhancement Combined Trial
