# Supplementary material for: Online Decision Support Tool for Personalized Cancer Symptom Checking in the Community (REACT): Acceptability, Feasibility, and Usability Study
Source: JMIR Cancer. 2018 Jul 4;4(2):e10073. doi: 10.2196/10073 (PMC6053613; doi:10.2196/10073)
Supplement: Multimedia Appendix 1 [file cancer_v4i2e10073_app1.pdf]

Multimedia Appendix 1

REACT open-ended questionnaire

1) Please tell us your gender.

a) Male
b) Female
c) Prefer not to say

2) Please tell us your age.

a) 30-39
b) 40-49
c) 50-59
d) 60-69
e) >=70

3) Have you been ever diagnosed with cancer?

a) Yes
b) No
c) Prefer not to say

4) What did you like/dislike about the website in general? Please write your answer in the text box below.

5) How could the website be improved? Please write your answer in the text box below.

6) How could the questionnaire be made more clear? Please write your answer in the text box below.

7) How could the questionnaire be improved? Please write your answer in the text box below.

8) What did you like/dislike about the risk presentation page? Please write your answer in the text box below.

9) Is there additional information that you would like to see displayed with your risk estimate? Please write your answer in the text box below.

10) We are trying the tool in community pharmacies and GP surgeries; where else would it be useful? Please write your answer in the text box below.

11) Would a tool like REACT be useful if available in:

a) Council Offices
b) Leisure Centres
c) Benefits Offices
d) Workplaces
e) With voluntary organisations (e.g. Age Concern)
